# Early Plant Development as a Systems-Level Trait: Integrating Omics, Artificial Intelligence, and Emerging Biotechnologies

**DOI:** 10.3390/plants15050787

**Published:** 2026-03-04

**Authors:** Abdallah S. Al-Sawa’eer, Ali Al-Samydai, Lama Odeh, Fatima Haj Ahmad, Renata Obekh, Yousef M. Abd Elqader, Anas Khaleel, Ahmad M. Al-Athamneh, Mariachiara Gabriele, Simonetta Cristina Di Simone, Claudio Ferrante, Luigi Menghini, Ahmed S. A. Ali Agha

**Affiliations:** 1 National Seeds Production Company, Amman 11183, Jordan; abdallahsawaeer10@yahoo.com (A.S.A.-S.); yousef.12alsatari@gmail.com (Y.M.A.E.); 2 Department of Pharmaceutics and Pharmaceutical Technology, Pharmacological and Diagnostic Research Centre, Faculty of Pharmacy, Al-Ahliyya Amman University, Amman 19111, Jordan; a.alsamydai@ammanu.edu.jo; 3 Department of Biology and Biotechnology, Faculty of Science, The Hashemite University, Zarqa 13133, Jordan; lamaodeh739@gmail.com (L.O.); renata.931931@gmail.com (R.O.); 4 Department of Biotechnology, Faculty of Agricultural Technology, Al-Balqa Applied University, Al-Salt 19117, Jordan; 5 Department of Clinical Pharmacy and Pharmacy Practice, Faculty of Pharmacy and Medical Sciences, University of Petra, Amman 11196, Jordan; anas.khaleel@uop.edu.jo; 6 Department of Nutrition, Faculty of Pharmacy and Medical Sciences, University of Petra, Amman 11196, Jordan; ahmad.alathamneh@uop.edu.jo; 7 Botanical Garden “Giardino dei Semplici”, Department of Pharmacy, “G. d’Annunzio” University “Chieti-Pescara”, Via dei Vestini n. 31, 66100 Chieti, Italy; mariachiara.gabriele@phd.unich.it (M.G.); simonetta.disimone@unich.it (S.C.D.S.); luigi.menghini@unich.it (L.M.); 8 Department of Pharmaceutical Sciences, School of Pharmacy, The University of Jordan, Amman 11942, Jordan

**Keywords:** seed germination, early seedling vigor, CRISPR-Cas, nanopriming, multi-omics, artificial intelligence

## Abstract

Seed germination and early seedling development are critical determinants of crop establishment, stress tolerance, and yield stability, yet these stages remain insufficiently integrated into contemporary crop improvement strategies. Recent advances across genome editing, microbiome-assisted seed treatments, nanotechnology-enabled priming, and artificial intelligence-guided phenotyping have generated substantial but fragmented insights into early developmental regulation. This review synthesizes recent advances across early plant development research. It demonstrates that seemingly diverse technologies converge on a limited set of regulatory control nodes, including abscisic acid–gibberellin balance, redox homeostasis, and root system architectural plasticity. By integrating evidence from molecular, microbial, physicochemical, and computational studies, early plant ontogeny is presented as a tunable regulatory state governed by quantitative thresholds rather than as a strictly predetermined genetic process. Advances in deep learning, reinforcement learning, and high-throughput phenotyping further enable the modeling and optimization of early developmental trajectories across genotype by environment contexts. Together, these insights establish early development as a programmable target for crop improvement and provide a mechanistic foundation for designing integrated interventions that enhance developmental uniformity, stress resilience, and yield stability across diverse agroecological systems.

## 1. Introduction

The early phases of plant ontogeny—comprising seed germination, seedling establishment, and the spatial organization of root–shoot architecture—are foundational to plant performance and agricultural productivity. These developmental transitions critically influence resource acquisition, stress resilience, and reproductive potential across diverse agroecological contexts [1]. The biophysical and biochemical coordination of these processes is governed by a complex interplay between intrinsic genetic programs and extrinsic environmental cues [2], including temperature, light, moisture, and soil composition [3].

Seeding traits such as mass, lipid composition, and embryo morphology directly modulate germination speed, emergence success, and seedling vigor. Concurrently, phytohormonal pathways—most notably involving abscisic acid (ABA), gibberellic acid (GA), and ethylene—regulate dormancy, reserve mobilization, and radicle emergence [4,5]. Given projected demands of over a 60% increase in global food production by 2050, early-stage developmental robustness has become a strategic target in crop improvement programs (FAO/UN) [6]. However, this critical ontogenetic phase is particularly susceptible to abiotic perturbations, most notably thermal and hydric stress, which disrupt the temporal dynamics of germination, compromise seedling morpho-uniformity, and attenuate early vegetative vigor [7,8]. These disruptions compromise stand establishment and downstream yield formation, particularly under resource limitation or environmental stress, highlighting the need to prioritize early developmental traits in breeding pipelines for abiotic stress resilience. The integration of phenotypic selection with molecular, genomic, and physiological indicators—particularly those associated with germination kinetics, root system ontogeny, and seedling vigor—has emerged as a critical framework for the development of cultivars adapted to increasing environmental variability [9,10]. This approach is essential for advancing climate-resilient crop ideotypes capable of maintaining developmental stability and productivity under fluctuating and suboptimal field conditions. Recent technological advances in high-throughput phenotyping, machine vision, and computational biology have transformed the study of early plant development [11]. Automated 2D and 3D imaging platforms—utilizing modalities such as magnetic resonance imaging (MRI) and laser scanning—now enable non-destructive, spatiotemporal analysis of root system architecture (RSA) and seedling growth dynamics under both controlled and field-like conditions [12]. Coupled with computational frameworks such as RootBox and CellSeT, these imaging outputs can be used to simulate mature RSA from seedling phenotypes, enhancing trait inference and scalability of early-stage selection protocols [13,14].

Complementary innovations in microfluidics have enabled in situ monitoring of germination and early seedling development at single-seed resolution under chemically and mechanically defined environments [15]. Moreover, the application of artificial intelligence (AI) and machine learning (ML) to plant phenotypic datasets has facilitated predictive modeling of genotype-by-environment (G × E) interactions, enabling automated trait extraction, pattern recognition, and digital twin construction for developmental forecasting in both model and crop species [11,16,17]. Therefore, this review critically synthesizes recent advances in the molecular, physiological, and computational understanding of early plant development, with a particular focus on seed germination, seedling establishment, and root–shoot architectural dynamics. Emphasis is placed on integrative strategies that combine high-resolution phenotyping, trait-based selection, and AI-driven modeling to enhance developmental precision and stress resilience. By consolidating insights across genomics, phenomics, and predictive systems biology, this work aims to inform the rational design of early-stage interventions for next-generation climate-resilient crop ideotypes.

## 2. Key Regulatory Components Governing Hormone-Responsive Germination

**Figure 1:**
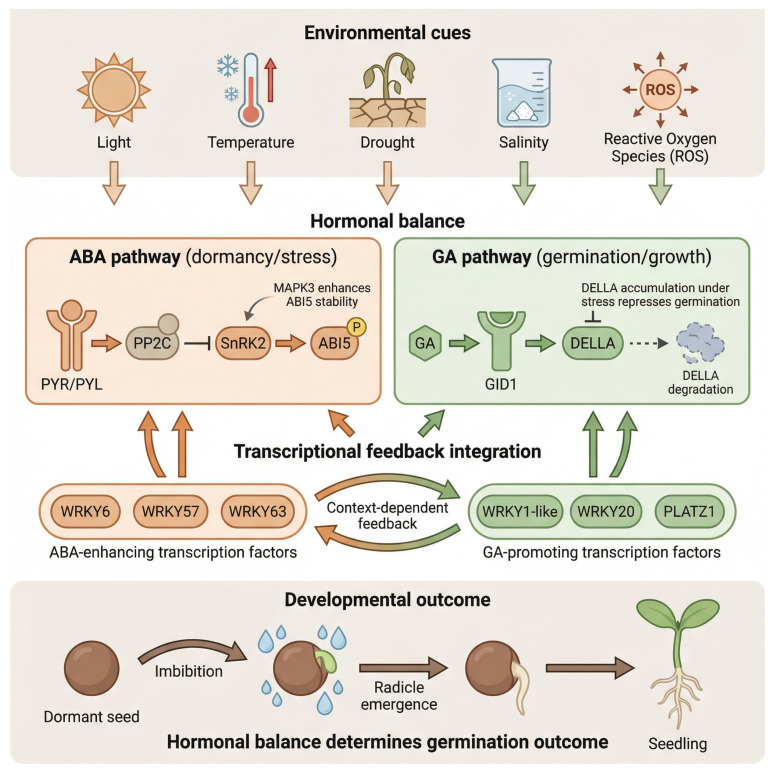
Integrated hormonal regulatory network governing seed germination in plants. Conceptual model illustrating how environmental cues—including light, temperature, drought, salinity, and reactive oxygen species (ROS)—converge on the core abscisic acid (ABA) and gibberellic acid (GA) signaling modules to regulate dormancy release, radicle emergence, and early seedling growth. The ABA pathway (PYR/PYL–PP2C–SnRK2–ABI5) and GA pathway (GID1–DELLA) are depicted with representative phosphorylation (P) and degradation events. Selected WRKY transcription factors and PLATZ1 provide transcriptional feedback integration between the antagonistic ABA and GA networks, thereby fine-tuning hormonal balance under environmental stress. Arrows indicate activation, T-bars denote inhibition, and “P” marks phosphorylation. The model is schematic and integrative rather than exhaustive.

Seed germination is a critical developmental checkpoint that integrates endogenous hormonal signals with exogenous environmental cues to ensure the successful transition from dormancy to autotrophic growth [18]. The regulatory landscape governing this process is dominated by the antagonistic interplay between abscisic acid (ABA), which promotes dormancy and stress resilience, and gibberellic acid (GA), which stimulates reserve mobilization and growth initiation [19]. A conceptual overview of this hormone-responsive regulatory architecture—illustrating how environmental cues converge on core ABA and GA signaling modules and are integrated through transcriptional feedback loops to control dormancy release, radicle emergence, and early seedling growth is presented in Figure 1. The components summarized in Table 1 represent a diverse array of transcription factors and signaling intermediates that modulate these hormonal pathways in a context-specific manner to optimize germination timing and seedling vigor.

In a plant developmental context, these components function within a hierarchical signaling framework encompassing hormone perception, signal transduction, transcriptional regulation, and the downstream execution of germination-associated physiological processes. At the core of dormancy maintenance and stress-induced germination repression lies canonical ABA signaling. ABA is perceived by PYR/PYL/RCAR receptors [46], which inhibit clade A PP2C phosphatases (e.g., ABI1 and ABI2), thereby releasing SnRK2 kinases (SnRK2.2, SnRK2.3, and OST1) [47]. Activated subclass III SnRK2 kinases (SnRK2.2/SRK2D, SnRK2.3/SRK2I, and SnRK2.6/SRK2E/OST1) act as primary regulators of ABA-dependent transcription under osmotic and drought stress. They coordinate downstream transcriptional programs by phosphorylating AREB/ABF transcription factors. Direct in vitro phosphorylation assays and mutant analyses show that these kinases phosphorylate and activate bZIP transcription factors of the AREB/ABF family—ABF1, ABF2/AREB1, ABF3, and ABF4/AREB2—which function as the dominant transcriptional regulators of ABA-induced gene expression [48,49]. Triple snrk2.2 snrk2.3 snrk2.6 mutants exhibit drastically reduced expression of ABA- and water stress-responsive genes, including LEA and PP2C genes, confirming that these kinases are essential for ABRE-dependent transcription under osmotic stress [49]. Phosphoproteomic and mechanistic studies demonstrate that SnRK2.2/2.3/2.6 phosphorylate AREB/ABF transcription factors at conserved RQxS/T motifs, thereby enhancing their DNA-binding and transactivation capacity in ABA signaling [50,51]. In vivo imaging and biochemical work demonstrate that OST1/SnRK2.6 phosphorylates ABF3 at Thr451, producing a 14-3-3 recognition site that stabilizes ABF3 and prolongs ABA-responsive transcription [52]. Recent studies also show that ABI5—another bZIP transcription factor functioning in ABA-mediated growth repression and seed desiccation tolerance—is phosphorylated and stabilized under ABA signaling, consistent with upstream activation by subclass III SnRK2s [53].

This ABA-dependent signaling module establishes a molecular brake on germination, ensuring seed survival under osmotic, salinity, or thermal stress, whereas gibberellin (GA) signaling promotes the execution phase of germination and early seedling growth. Bioactive GA binds to its soluble receptor GID1 (GIBBERELLIN INSENSITIVE DWARF1), forming a GA–GID1 complex that interacts with DELLA repressors (e.g., RGL2, GAI, and RGA) and recruits the SCF^SLY1/GID2 E3 ubiquitin ligase complex, leading to the ubiquitin-mediated proteasomal degradation of DELLA proteins and activation of GA-responsive transcription [54,55]. This removal of DELLA repression permits transcription of cell wall-loosening and metabolic genes, facilitating embryonic axis elongation and radicle protrusion, key steps in seed germination [56]. Under stressful or low-GA conditions, DELLA accumulation restrains GA-driven growth and reinforces ABA signaling, partly by promoting ABI5 activation and ABA biosynthesis, thereby maintaining seed dormancy [57]. Recent work also suggests that GA can enhance autophagic degradation of DELLA proteins through GID1–ATG8 interactions, complementing the canonical ubiquitin–proteasome pathway to fine-tune growth activation under stress [55]. The antagonism between abscisic acid (ABA) and gibberellin (GA) during seed germination operates through multiple regulatory layers. At the metabolic level, transcriptional control of ABA biosynthetic genes (NCED2, NCED5, NCED9) and GA biosynthetic genes (GA20ox, GA3ox) fine-tunes the hormonal balance between dormancy and germination. For example, high temperature or salinity stress elevates NCED expression while repressing GA20ox and GA3ox, increasing ABA levels and inhibiting germination [58,59]. Conversely, signals that favor germination—such as melatonin or nitrate—downregulate NCED and ABI5 while upregulating GA20ox and GA3ox, reducing ABA content and enhancing GA biosynthesis [60,61]. Collectively, WRKY transcription factors exhibit pronounced regulatory plasticity, functioning as either positive or negative modulators of germination depending on species, tissue context, and stress intensity. For instance, GhWRKY6-like and TaWRKY24 enhance germination and root elongation under drought or salinity through coordinated ABA–ROS regulation [24,32], whereas GhWRKY17 intensifies ABA sensitivity and ROS accumulation, thereby inhibiting germination under salt and drought stress [29]. Post-translational regulation further sharpens hormonal control during early ontogeny. MAPK3-mediated phosphorylation of ABI5 stabilizes the protein and promotes its nuclear localization, reinforcing ABA-dependent transcription during osmotic stress [22]. Taken together, hormone-responsive germination is governed by an integrated regulatory network in which ABA–GA antagonism, transcription factor plasticity, post-translational reinforcement, and ROS-mediated execution processes converge on shared molecular effectors. This systems-level organization provides multiple leverage points for breeding, genome editing, and seed-based biotechnological interventions aimed at optimizing germination timing and early seedling vigor under fluctuating environmental conditions.

## 3. Biotechnological Innovations for Trait Enhancement

### 3.1. CRISPR/Cas-Based Modulation of Dormancy, Stress, and Root Traits

**Figure 2:**
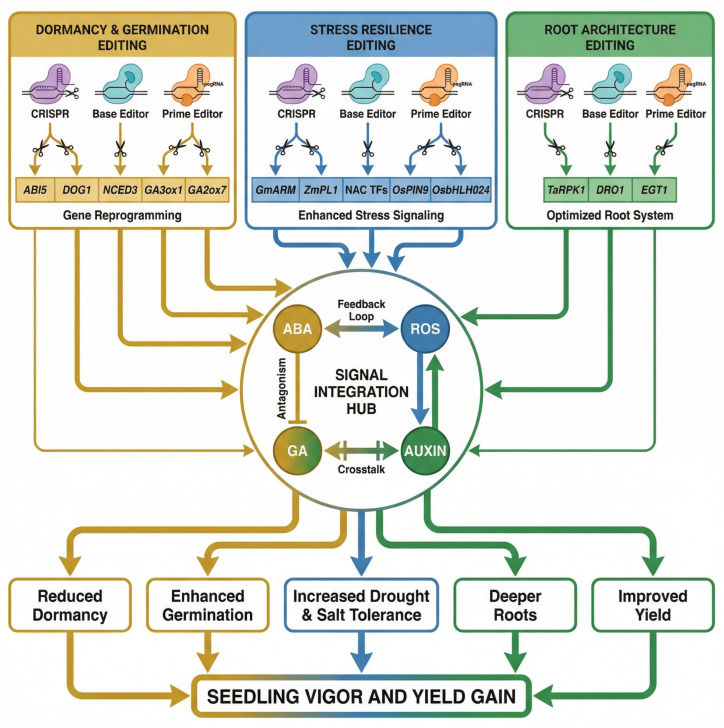
Mechanistic framework of CRISPR/Cas-mediated regulation of seed and seedling traits. Genome editing of key dormancy (ABI5, DOG1, NCED3, GA3ox1, GA2ox7), stress (GmARM, ZmPL1, NAC TFs, OsPIN9, OsbHLH024), and root genes (TaRPK1, DRO1, EGT1) reconfigures ABA–GA–ROS–auxin signaling networks. The integrated hub coordinates germination, stress tolerance, and root plasticity, illustrating how multiplex CRISPR edits drive systems-level enhancement of seedling vigor and yield.

CRISPR/Cas genome editing has emerged as a powerful tool to precisely modify seed and seedling traits that were previously difficult to fine-tune through conventional breeding [62]. Unlike transgenic approaches, CRISPR enables targeted changes in native genes—for example, knocking out inhibitors or altering hormone regulators—to enhance germination, stress resilience, and root development. Recent innovations such as base editors, prime editing, and promoter engineering have expanded the CRISPR toolkit, enabling subtle allele substitutions and refined regulatory control of trait-associated genes [63]. Recent innovations such as base editors and prime editing expand this toolkit, allowing subtle allele substitutions and refined control over trait-associated genes [62]. Collectively, these advances enable precise modulation of key seed traits and expand the scope of crop improvement strategies. CRISPR/Cas-mediated improvements in seed germination and early seedling vigor frequently target hormonal and redox signaling hubs, particularly those involving abscisic acid (ABA) perception, gibberellin (GA) biosynthesis, and oxidative stress regulation. Recent genome-editing studies have demonstrated that knocking out ABA receptor genes in soybean using CRISPR/Cas9 reduces ABA sensitivity and promotes germination under stress conditions [64]. Similarly, CRISPR-induced mutations in ABI5 regulators in rapeseed enhanced drought resistance and early root development by modulating ABA-driven gene networks [65]. In the gibberellin pathway, targeted editing of GA3ox1 in barley optimized GA levels to improve coleoptile elongation and seedling emergence under drought stress [66], while CRISPR knockouts of OsGA2ox7 in rice enhanced germination under salt stress by preventing GA degradation [67]. In addition, CRISPR-mediated editing of redox and transcriptional regulators such as HY5 in Chinese cabbage has been shown to mitigate reactive oxygen species accumulation and improve early growth under stress [68]. Another notable application is using CRISPR to adjust seed dormancy duration and germination timing. For instance, in barley, two quantitative trait loci, Qsd1 and Qsd2, control grain dormancy—a critical balance between preventing pre-harvest sprouting and enabling uniform malting and germination. Hisano et al. [69] created CRISPR/Cas9 knock-outs of Qsd1 and Qsd2 (both singly and in combination) to dissect their roles [69]. Germination assays revealed that mutations in either gene delayed seed germination, and double mutants showed strongly enhanced dormancy, accumulating higher abscisic acid (ABA) levels than wild type [69]. Interestingly, the prolonged dormancy of qsd2 mutants was partly suppressed if Qsd1 was also mutated, indicating an epistatic interaction. These results demonstrated that normal, rapid germination in barley requires both genes, and that combining loss-of-function alleles can “tune” the dormancy period. Such insights are invaluable for designing cereal varieties. By editing dormancy genes, breeders can achieve optimal sprouting resistance without sacrificing germination uniformity. Indeed, the barley CRISPR mutants now provide germplasm to further study and engineer grain dormancy traits [69]. Similarly, DOG1 and NCED3—key regulators of ABA synthesis and seed dormancy—are now being targeted using dual-function editing systems to optimize sprouting resistance without compromising germination uniformity [70]. Early plant development is often limited by abiotic stresses (drought, salinity, heat). CRISPR is accelerating the development of stress-resilient genotypes by enabling the removal of sensitivity factors and the activation of latent tolerances. A clear example is in soybean (Glycine max), where an ABA signaling repressor gene GmARM was knocked out via CRISPR/Cas9. The edited soybeans exhibited improved tolerance to multiple stresses, including alkali salt stress and even resistance to a root rot pathogen, compared to wild-type plants [71]. This suggests that GmARM normally constrains stress responses, and its disruption frees the plant to mount a stronger defense (likely by elevating ABA responsiveness). In maize, CRISPR was used to investigate a stress-inducible gene ZmPL1 (encoding a phylloplanin-like protein). Interestingly, maize lines with CRISPR-induced loss-of-function in ZmPL1 outperformed controls under drought conditions: they showed higher seed germination and seedling survival rates, lower oxidative damage (less MDA and ROS accumulation), and increased activity of antioxidant enzymes [71]. The edited plants also up-regulated various drought-responsive genes, indicating a primed stress defense state. These findings revealed ZmPL1 as a negative regulator of drought tolerance in maize, so its removal confers robust resistance. Additional targets such as OsbHLH024 and OsPIN9 in rice have conferred salt and chilling tolerance, respectively, by reprogramming transcriptional networks and maintaining ROS homeostasis [72,73]. The knockout of NAC transcription factors in rice and wheat has improved germination efficiency and early root vigor under salinity and drought conditions [74,75]. Taken together, these findings indicate that CRISPR-mediated editing of regulatory genes frequently produces network-level phenotypic effects because many of the targeted loci function within interconnected hormone, redox, and developmental pathways governing early plant growth. Because these pathways are intrinsically integrated during early ontogeny, edits targeting components of these networks can converge at shared regulatory hubs where ABA–GA antagonism, ROS feedback, and auxin signaling intersect to coordinate germination, stress responses, and root system development. This interconnected framework is illustrated in Figure 2, which maps how targeted genome edits converge on a central ABA–GA–ROS–auxin integration hub that coordinates these processes to enhance seedling vigor and yield.

More generally, numerous studies confirm that CRISPR-based knockouts of yield–penalizing stress genes (e.g., those controlling excessive stomatal opening or senescence) can produce crops that endure water deficit, salinity, and temperature extremes [62]. However, stress-tolerance edits must be evaluated for trade-offs. For example, deleting genes involved in stomatal development can conserve water but may impair CO_2_ intake and growth. In foxtail millet, CRISPR/Cas9 mutagenesis of the stomatal density gene SiEPF2 indeed reduced drought stress via lower stomatal density, but unexpectedly also reduced yield by altering panicle morphology [76]. This underscores the need for precision editing strategies (e.g., tissue-specific or inducible CRISPR) to balance stress protection with growth—an area where advanced CRISPR variants and guided selection can play a role. Another frontier is using CRISPR to redesign root traits for better seedling establishment and resource uptake. Roots determine how well a young plant acquires water and nutrients, especially under stress, so architectural traits like root depth, branching, and gravitropic angle are prime targets for improvement. Genome editing has demonstrated that we can indeed push root traits beyond their natural limits. In wheat, for example, researchers edited a gene (TaRPK1) suspected to influence root architecture. Using dual-gRNA CRISPR constructs delivered via Agrobacterium, they generated wheat lines with targeted mutations in TaRPK1 homologs [77]. The TaRPK1-edited wheat showed dramatic changes in root system architecture (RSA): notably deeper and longer roots, greater root volume and surface area, but reduced lateral root number and shallower root angle, compared to unedited plants [77]. These RSA changes had positive knock-on effects—edited lines developed more productive tillers and achieved higher total grain weight per plant than wild type. In essence, CRISPR-created root modifications improved wheat yield by facilitating better soil foraging (even though a slight reduction in spike length per head was observed). Similarly, modifying Ospmei12, a gene involved in cell wall biosynthesis, improved root structure and conferred tolerance to heavy metal stress and hormonal imbalance [78]. This result validates the long-sought hypothesis that deeper roots can enhance yield under field conditions. Similarly, gene targets like DRO1 (which controls root gravitropic angle) in rice and EGT1/EGT2 (enhanced gravitropism genes) in cereals have been highlighted as promising candidates for editing [77]. Natural allelic variants of DRO1 make rice roots grow deeper, improving drought avoidance, and now CRISPR could be used to introgress or mimic these alleles in elite cultivars. More broadly, CRISPR is enabling ideotype breeding for roots, e.g., creating crops with steeper angles or more finely branched roots to capture nutrients efficiently without penalizing above-ground growth [77]. These findings illustrate how multiplex CRISPR platforms, guided by systems biology approaches, are enabling the coordinated reprogramming of dormancy, stress response, and root development networks [79].

Importantly, the translational relevance of regulatory-node editing has progressed beyond controlled experimental systems toward regulatory clearance and commercial cultivation. A high-oleic soybean variety generated through targeted genome editing—via small nucleotide deletions disrupting the endogenous fatty acid desaturase genes FAD2 and FAD3—was determined by the United States Department of Agriculture to fall outside its regulatory authority under the “Am I Regulated?” framework, thereby enabling accelerated market entry and subsequent commercialization within the U.S. agricultural supply chain and representing one of the earliest large-scale deployments of a gene-edited commodity crop [80]. Similarly, CRISPR/Cas9-mediated disruption of the autoinhibitory C-terminal domain of endogenous glutamate decarboxylase genes (SlGAD2 and SlGAD3) enabled large increases in γ-aminobutyric acid (GABA) accumulation in tomato fruits, establishing a precise metabolic-pathway engineering strategy for functional trait enhancement. Building on this molecular framework, Japan implemented a product-based regulatory notification system for genome-edited crops, under which a high-GABA tomato developed via targeted genome editing was notified and subsequently commercialized without transgene integration, representing one of the first market-authorized genome-edited food products derived from endogenous pathway modulation [81]. In parallel, controlled flooding experiments in the terrestrial species Alternanthera philoxeroides demonstrate that tolerance to water-associated stress does not rely on increased root biomass investment per se, but rather on efficiency-oriented anatomical adaptation. Under dissolved oxygen deficiency, root-flooded plants reduced biomass allocation to roots while simultaneously increasing root efficiency through enlargement of aerenchyma channel diameter, thereby enhancing internal oxygen conductance and sustaining nutrient uptake despite hypoxic conditions. These findings provide direct experimental evidence that stress resilience under flooding can be achieved through structural optimization of existing organs rather than compensatory growth responses [82]. Collectively, these cases indicate that developmental and hormone-associated genetic interventions are increasingly being incorporated into applied breeding frameworks rather than remaining confined to proof-of-concept experimentation.

In summary, CRISPR/Cas-based approaches are driving a new era of seed trait optimization. By directly tweaking endogenous genes that control dormancy, stress responses, and root development, researchers have shown improvements in germination behavior, seedling survival, and yield that would be difficult to achieve with conventional methods alone. Ongoing advances in refined Cas enzymes, transcriptional activation/repression systems, and base-editing tools are poised to further increase the specificity and scope of trait engineering [63].

### 3.2. Microbiome-Assisted Germination and Root-Soil Interaction Engineering

Seeds are not sterile vessels; they carry complex communities of microorganisms (the seed microbiome) that can profoundly influence germination and early growth. Recent studies have illuminated the critical symbiosis between seeds and their microbiomes in promoting plant vigor, stress tolerance, and soil adaptation [83,84]. This has given rise to next-generation strategies where beneficial microbes—either naturally associated or introduced—are leveraged to enhance seed performance. By engineering the plant microbiome, scientists aim to improve traits like germination rate, seedling establishment, and root development through biological means rather than genetic modification of the plant itself [83]. Seeds harbor endophytic bacteria and fungi inside their tissues and on their surfaces, which are often passed from the parent plant via the seed (vertical transmission) [83,84]. Upon germination, many of these seed-borne microbes become the pioneers of the seedling’s root microbiome, even surpassing indigenous soil microbes in colonizing the rhizosphere [83]. For example, a recent study on wheat used high-resolution sequencing to track microbiome assembly and found that seed-borne bacteria dominated the rhizosphere community, outcompeting soil-derived microbes [83]. These seed-origin microbes (coined “seed-borne rhizosphere bacteria”, SbRB) were enriched in genes for utilizing specific seed/root exudates (sugars, etc.) and for mutualistic interactions like cross-feeding, giving them a competitive edge in the young root zone. In essence, the seed comes pre-packaged with microbes optimized to jump-start seedling growth—they can digest seed exudates and even help each other grow (niche partitioning and facilitation) [83]. This finding shifts our understanding of early plant establishment: rather than roots simply recruiting microbes from soil, the seed’s own microbiota significantly shapes the initial rhizosphere. Such knowledge opens the door to manipulating seed microbiomes to favor beneficial species that support germination and suppress deleterious ones. Beneficial seed-associated microbes influence the abscisic acid (ABA)–gibberellin (GA) balance via enzymatic and signaling mechanisms that reshape the hormonal environment around the embryo. For example, Priestia megaterium PH3 colonization in peanut reduced ABA sensitivity by upregulating ABA catabolic genes (AhCYP707A1) and stimulated GA biosynthesis through activation of GA20ox and GA3ox, collectively accelerating germination under salt stress [84]. Similarly, the fungal endophyte Marquandomyces marquandii enhanced Chinese cabbage seed germination by increasing GA and IAA content, lowering ABA levels, and upregulating stress-resistance gene networks [85].

**Figure 3:**
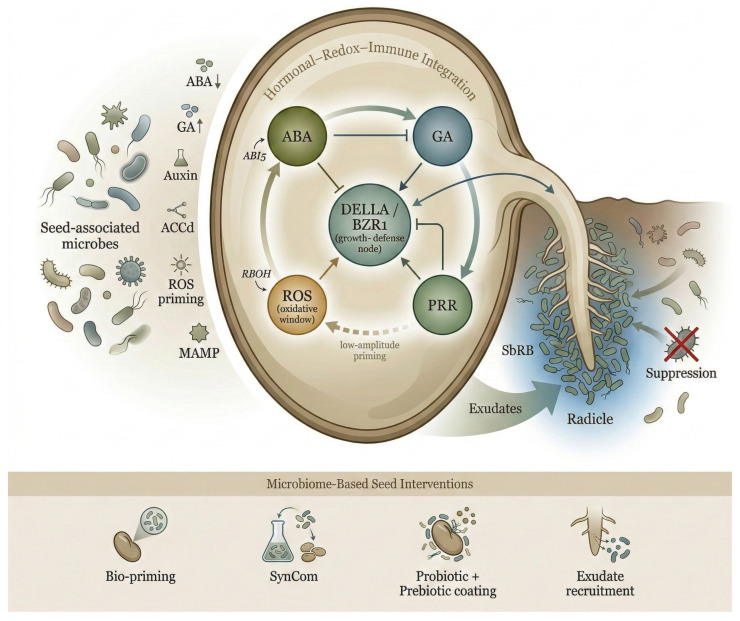
Systems model of microbiome-mediated seed germination and early root establishment. Seed-associated microbes modulate the embryonic hormonal–redox–immune network by reducing abscisic acid (ABA), stimulating gibberellin (GA), producing auxins, expressing ACC deaminase (ACCd), priming reactive oxygen species (ROS), and activating pattern-recognition receptors (PRRs) through microbe-associated molecular patterns (MAMPs). These inputs converge on an ABA–GA–ROS–PRR regulatory circuit involving ABI5, DELLA/BZR1 (growth–defense node), and respiratory burst oxidase homolog (RBOH), coordinating dormancy release, immune priming, and radicle elongation. Root exudates enrich seed-borne rhizosphere bacteria (SbRB), supporting early microbiome assembly and pathogen suppression. Microbiome-based seed interventions—including bio-priming, synthetic consortia (SynCom), probiotic and prebiotic seed coatings, and exudate-guided recruitment—exploit these mechanisms to enhance seedling establishment and stress resilience.

At the biochemical level, endophytic and rhizospheric microbes often express ABA-degrading or GA-mimicking enzymes, such as CYP707A-like oxygenases or GA20-oxidase analogs, which shift the ABA:GA ratio toward germination [86]. Concurrently, many of these microbes secrete tryptophan-derived auxins or ACC deaminase, alleviating ethylene inhibition and facilitating embryo cell expansion. Microbial modulation of redox homeostasis constitutes a parallel control layer. Beneficial microbes prime the reactive oxygen species (ROS) system by activating antioxidant enzymes such as SOD, CAT, and GR, maintaining ROS concentrations within the “oxidative window” permissive for germination [87]. Controlled ROS pulses activate NADPH oxidases (RBOHD/F), which act as apoplastic signal amplifiers linking environmental cues to hormonal pathways [88]. These redox bursts promote GA synthesis and weaken ABA signaling via redox-sensitive regulation of ABI5 and DOG1, integrating oxidative and hormonal control during dormancy release [89]. In parallel, microbe-associated molecular patterns (MAMPs) such as flagellin and chitin fragments activate pattern-recognition receptors (PRRs), including FLS2, CERK1, and FERONIA (FER), triggering low-amplitude MAPK3/6 cascades that intersect with hormonal pathways [90]. This mild immune priming modulates growth–defense cross-talk hubs, notably DELLA and BZR1, which integrate gibberellin and brassinosteroid signaling with immune output [91,92]. Through this interplay, beneficial microbes initiate a “controlled defense readiness” state that promotes vigor without incurring growth penalties [93]. Beneficial seed-associated microbes influence the ABA–GA balance via enzymatic and signaling activities, reshape oxidative dynamics, and fine-tune immune cross-talk through pattern-recognition receptors. Through these coordinated effects, microbial partners establish a regulatory interface that couples hormonal, redox, and defense pathways to promote germination and stress resilience. This integrated mechanism is summarized in Figure 3, which depicts how seed-borne and rhizospheric microbes influence ABA–GA–ROS–MAMP signaling and how these processes translate into practical microbiome-engineering strategies for crop establishment.

Diverse microbial taxa have been identified that enhance seed germination and seedling development. Many plant growth-promoting rhizobacteria (PGPR) or fungi produce phytohormones, mobilize nutrients, or protect against pathogens. Seed endophytes in various crops are known to fix nitrogen, solubilize phosphorus, synthesize auxins and other growth hormones, and generate antimicrobial compounds, thereby boosting early plant growth and health [84]. A notable example comes from a study on Astragalus mongholicus seeds, where researchers compared the microbiomes of germinated versus ungerminated seeds [84]. Germinated seeds hosted a far more diverse and probiotic-rich microbial community—genera like Pseudomonas and Pantoea were enriched—whereas ungerminated seeds were dominated by Fusarium (a pathogenic fungus). Metagenomic analysis revealed that the microbiome in germinated seeds carried more genes for pathogen inhibition (e.g., antibiotic production) and cellulose degradation (breaking down seed coat components), functions presumably aiding the germination process. Indeed, the presence of Fusarium was associated with seeds failing to germinate, while seeds that did germinate had microbes actively suppressing Fusarium. Through follow-up inoculation experiments, the researchers confirmed that introducing certain bacteria (e.g., Paenibacillus species) could suppress the Fusarium pathogen and produce cellulases, thereby significantly improving seed germination rate and seedling vigor. This demonstrates a causal link: a healthy seed microbiome can actively fend off seed-borne pathogens (which often cause seed rot or damping-off) and help break physical dormancy (by softening the seed coat via cellulose breakdown), leading to more successful germination [84]. Field crops show similar patterns; for instance, natural seed-borne Bacillus and Trichoderma strains are known to colonize seedlings and protect them against soil pathogens that cause wilt and root diseases. By capitalizing on such microbes, we can enhance early plant immunity and growth without genetic changes to the plant itself. To enhance seed germination and early development, scientists are engineering the seed and root microbiome through approaches like bio-priming with beneficial microbes (e.g., Azospirillum, Pseudomonas), breeding for favorable seed endophytes, and introducing synthetic (SynCom) or natural (NatCom) microbial communities [94,95]. These strategies aim to establish stable, beneficial microbiomes in complex soils. Additionally, manipulating root exudates to attract advantageous microbes, or using seed coatings with prebiotics and signaling molecules, supports the concept of the plant holobiont—treating the plant and its microbiome as one system. Importantly, microbiome-mediated effects are most pronounced during the early germination and seedling establishment window, when hormonal, redox, and immune thresholds are highly plastic. These microbiome-assisted innovations enhance seedling vigor, stress tolerance, and reduce reliance on chemical inputs, marking a shift toward biologically integrated agriculture.

Evidence from rigorously designed multi-location, multi-season field trials supports the translational potential of microbiome-assisted strategies for crop establishment and productivity. Inoculation with *Azospirillum brasilense* resulted in statistically significant and reproducible yield increases in both maize and wheat across distinct agroecological regions in southern Brazil when evaluated under regulatory-grade field protocols. Grain yield improvements ranged from approximately 16–30% in maize and 9–18% in wheat, with consistent performance of selected strains across independent sites and growing seasons [96]. Importantly, these gains were achieved under low starter nitrogen inputs and were attributed primarily to enhanced root development and increased uptake of multiple macro- and micronutrients, rather than direct biological nitrogen fixation, thereby demonstrating that targeted selection of plant-growth-promoting bacterial strains can translate mechanistic plant–microbe interactions into agronomically meaningful outcomes under real-world cultivation conditions [96].

### 3.3. Nanotechnology-Enabled Seed Priming for Enhanced Germination and Stress Tolerance

**Figure 4:**
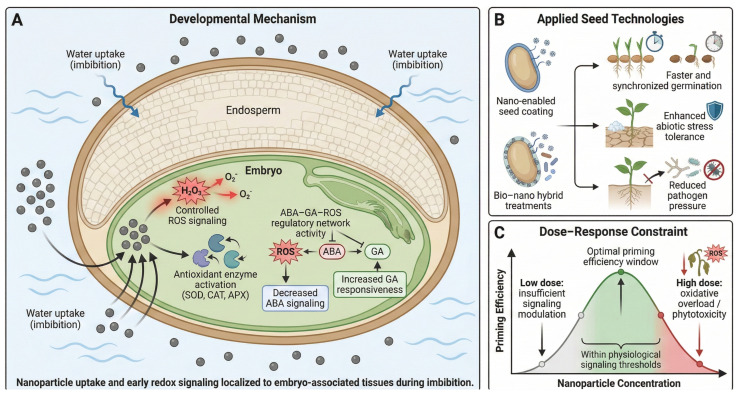
Nanoparticle-mediated seed priming: developmental mechanism, applied technologies, and dose–response constraint. (**A**) During imbibition, nanoparticles interact with the seed surface and are illustrated as entering embryo-proximal tissues, where they transiently modulate redox homeostasis. This includes controlled ROS signaling (H_2_O_2_, O_2_^−^), activation of antioxidant enzymes (SOD, CAT, APX), and modulation of ABA–GA–ROS regulatory network activity, characterized by decreased ABA signaling and increased GA responsiveness. The endosperm is depicted as anatomically present but not as a primary site of nanoparticle uptake or early signaling at this stage. (**B**) Nano-enabled seed coatings and bio–nano hybrid treatments translate these physiological effects into improved germination performance, enhanced abiotic stress tolerance, and reduced pathogen pressure. (**C**) Effective nanopriming operates within an optimal concentration-dependent “priming efficiency window,” bounded by insufficient signaling at low doses and oxidative overload or phytotoxicity at high doses.

Nanopriming represents a cutting-edge approach in seed technology, utilizing engineered nanoparticles (NPs) to improve germination rates, seedling vigor, and resilience to abiotic and biotic stresses. Unlike traditional priming, which primarily involves hydration, nanopriming exploits the nanoscale size, high surface area, and reactivity of particles to modulate seed physiology at the cellular and molecular levels [97]. Mechanistically, nanopriming enhances water imbibition by creating nanopores in the seed coat and induces controlled oxidative priming through low-level reactive oxygen species (ROS) production [97]. These effects stimulate antioxidant defenses, activate germination enzymes (e.g., α-amylase), and upregulate aquaporins, leading to accelerated and synchronized germination [97]. Some nanoparticles, such as selenium (SeNPs), penetrate seed cells and localize near plastids, modulating hormonal and metabolic pathways that promote early seedling development [98]. Numerous case studies across crops such as tomato, rice, pea, and canola demonstrate the efficacy of various NPs, including Fe_3_O_4_, CaO, Se, and AgNPs, in enhancing drought tolerance, nutrient status, biomass accumulation, and disease resistance. For instance, Fe_3_O_4_ NPs improved drought resilience in Pisum sativum, while CaO NPs enhanced germination and seedling vigor in canola [98]. SeNPs conferred both growth and antifungal benefits in rice and tomato, e.g., ~72.9% protection against *Phytophthora* infestans and increased chlorophyll content [98]. Similarly, Se-loaded mesoporous silica improved tomato germination and exhibited antifungal activity against *Botrytis cinerea* [98]. AgNPs applied to pea seeds reduced fungal infection by over 70–90% without impairing germination [99]. Building on these successes, researchers are developing smart seed coatings that incorporate NPs for responsive release upon hydration [97,98]. Hybrid strategies combining nanopriming with bio-priming—such as encapsulating beneficial microbes with clay or polymer-based nanocarriers—are also being explored to synergize microbial colonization with nanoparticle-induced enhancements [98]. Additionally, carbon-based nanomaterials like graphene quantum dots are under investigation for their potential to deliver germination-stimulating molecules and modulate developmental signaling pathways [98]. At a systems level, many nanopriming-induced responses converge on the same abscisic acid (ABA)–gibberellin (GA)–reactive oxygen species (ROS) regulatory nodes that govern dormancy release and early seedling growth. Rather than acting as independent growth drivers, nanoparticles primarily function as modulators of endogenous signaling thresholds, sensitizing seeds to favorable germination cues while reinforcing stress-adaptive buffering during early ontogeny. Nanopriming acts as a transient developmental reprogramming event that modulates the same hormonal and redox nodes controlling dormancy release and early seedling vigor. These mechanisms are summarized in Figure 4, which illustrates how nanoparticles influence water uptake, oxidative and hormonal signaling, and how these processes are translated into practical nanopriming systems. As shown, the initial nanoparticle–seed interactions trigger controlled ROS generation and ABA–GA re-balancing within a narrow “oxidative window,” producing a short-lived but highly effective priming state that enhances germination and stress readiness.

Dose optimization is critical in nanopriming applications, as excessive concentrations of nanoparticles (NPs) can induce phytotoxicity, oxidative stress, and impaired germination. Importantly, nanopriming acts predominantly as a transient pre-conditioning stimulus during seed imbibition and early metabolic activation, rather than as sustained nanoparticle exposure throughout plant development, distinguishing it from foliar or soil-based nanomaterial applications. Several crops, including barley, stevia, and rapeseed, demonstrate a threshold-dependent response, wherein low NP doses enhance physiological performance while higher concentrations result in cellular damage [97,98,100,101]. For instance, selenium nanoparticles (SeNPs) at ~28 ppm promoted rapeseed development, but higher doses were deleterious [98]. While many NPs employed in nanopriming—such as silica and iron oxide—are biodegradable or naturally abundant in soils, their nanoscale bioavailability and long-term ecological effects warrant further investigation [97,98]. Overall, nanopriming offers a promising, multifaceted platform for enhancing seed performance under suboptimal conditions, aligning with the goals of precision agriculture and sustainable crop production.

Recent literature indicates that nano-enabled agricultural inputs are beginning to be evaluated under application-relevant agronomic conditions rather than remaining limited to laboratory-scale proof-of-concept studies. Consistent with broader advances in polymer and nanomaterial functionalization—where surface engineering is used to regulate stability, interaction, and release behavior—nano-fertilizer systems, including nano-urea formulations, have been examined in field or semi-field trials for their potential to improve nutrient-use efficiency and sustain crop productivity while reducing overall fertilizer inputs, without implying regulatory approval or large-scale commercialization [102].

In conclusion, nanotechnology offers a novel toolkit to improve seed traits and early plant development in ways conventional methods cannot. By physically priming seeds at the nanoscale, we can achieve faster germination, uniform emergence, and seedlings that are pre-conditioned to withstand stresses. Table 2 summarizes key examples of nanopriming and their outcomes.

Collectively, the data shown in Table 2 reveal that nanoparticle-mediated seed priming exerts crop-specific and nanoparticle (NP)-dependent effects that extend beyond simple enhancements in germination. Iron-based nanomaterials (e.g., Fe_3_O_4_, Fe_2_O_3_) have consistently stimulated root elongation, chlorophyll biosynthesis, and antioxidant defense in crops like wheat and flax, particularly in legumes and cereals. These effects are likely mediated through improved micronutrient availability and enhanced redox balance during early development, although the exact molecular pathways remain to be fully elucidated [113,114]. In contrast, selenium and silver nanoparticles exhibit dual functionality—enhancing seed germination while contributing to biotic and abiotic stress tolerance. This is most likely mediated through modulation of reactive oxygen species (ROS) levels and upregulation of antioxidant enzymes such as superoxide dismutase (SOD), catalase (CAT), and glutathione peroxidase (GSH-PX), which help maintain cellular redox balance under stress conditions [115,116]. In the case of silver nanoparticles, seed priming has also been shown to reduce pathogen load and enhance disease resistance, possibly by priming metabolic pathways involved in stress-related secondary metabolites [99]. Zinc-based systems (ZnO, ZnS) appear particularly effective in crops like rice, wheat, and moringa, where they enhance nutrient uptake and translocation, increase chlorophyll content, and boost the expression of antioxidant and metabolic enzymes. These enhancements suggest a role in modulating physiological and biochemical processes critical for early seedling vigor and stress mitigation [104,117,118]. Carbon-based nanomaterials, such as multi-walled carbon nanotubes (MWCNTs), have been proposed in limited studies to affect water uptake via physical modulation of membrane properties, potentially involving aquaporin upregulation. However, direct evidence for this mechanism in crop systems remains scarce, and further research is needed to substantiate this claim. Despite these promising results, several knowledge gaps persist. A major limitation is the lack of standardized reporting for key NP characteristics such as hydrodynamic size, zeta potential, and aggregation behavior, which affects reproducibility and hampers mechanistic understanding. Additionally, very few studies assess long-term environmental impacts, soil fate of nanoparticles, or transgenerational effects on seed quality. To address this, we propose the development of a ‘Priming Efficiency Index’ (PEI)—a composite metric integrating germination rate, vigor index, biochemical markers, and crop-specific yield outcomes—to standardize efficacy assessments across studies. While no study has yet formalized such an index, several have highlighted the need for standardized, integrative metrics to improve comparability and reproducibility [119].

## 4. Bioinformatics and Omics-Driven Trait Discovery

**Figure 5:**
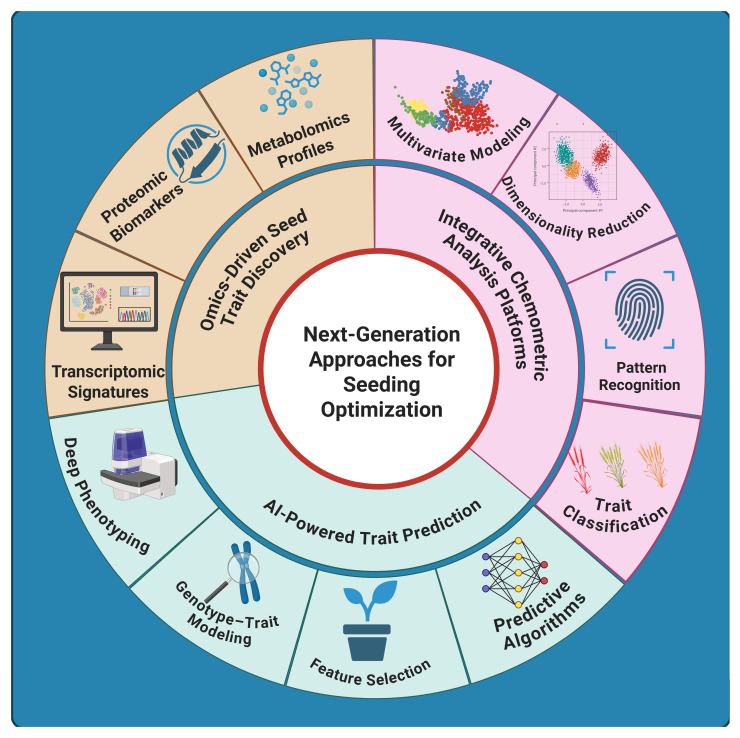
Integrated framework illustrating the convergence of omics-driven trait discovery, chemometric modeling, and AI-powered prediction in next-generation strategies for optimizing plant seeding and early development.

Modern omics technologies—genomics, transcriptomics, proteomics, metabolomics, among others—are revolutionizing the discovery of biomarkers and regulatory mechanisms underlying seed germination and early vigor. High-throughput analyses enable researchers to pinpoint molecular signatures (genes, proteins, metabolites) [120] that could distinguish high-vigor seeds or stress-resilient seedlings from their lower-performing counterparts. By integrating these multi-omics data with advanced bioinformatics and AI tools, it is now possible to build predictive models of seed performance and uncover complex gene networks that drive early development [121]. The integration of bioinformatics, omics, and artificial intelligence has reshaped how early plant traits are predicted and engineered. Figure 5 provides an overview of the multidimensional framework addressed in this review, illustrating how omics-based trait discovery, chemometric modeling, and AI-guided prediction converge to support next-generation strategies for seeding optimization.

Genome-wide association studies (GWAS) and transcriptome profiling have revealed numerous genetic loci and gene expression patterns linked to seed vigor traits. For example, a GWAS in rice identified OsOMT, encoding a 2-oxoglutarate/malate translocator, as a key gene regulating germination rate [122]. Knocking out OsOMT reduced germination and seedling growth, due to impaired amino acid, sugar, and energy metabolism in mutant seeds [122]. Likewise, other rice GWAS have uncovered vigor-associated genes such as OsCDP3.10 (a cupin-domain protein) and a cytochrome b5 involved in reserve mobilization [122]. In *Medicago truncatula*, integrating GWAS with RNA-seq led to the discovery of MtMIEL1 as a regulator of germination plasticity under maternal heat stress—a candidate validated by cross-species experiments [123]. Comparative transcriptomic studies also shed light on vigor differences, such as in an alpine plant (*Rheum pumilum*). RNA-seq across germination stages (coupled with co-expression network analysis) revealed that rapid germination at high altitudes is enabled by the concerted upregulation of six hormone pathways (ABA, GA, auxin, etc.) and key transcription factors (e.g., BZIP, PLATZ, WRKY families) [124]. Similarly, in quinoa, transcript profiling of two cultivars with contrasting seed storability (after controlled aging) showed that the aging-sensitive line had hundreds of stress-responsive genes perturbed, notably in flavonoid biosynthesis, tricarboxylic acid (TCA) cycle, and terpenoid pathways, whereas the aging-tolerant line maintained stable expression of carbon metabolism genes [125]. This indicates that preserving core metabolic functions (glycolysis, TCA cycle) while limiting stress-induced secondary metabolite disruptions is a hallmark of high-vigor seeds [125]. Seed proteome analyses have identified protein biomarkers and pathways correlated with vigor and stress resilience. A proteome-wide study in sugar beet detected 759 seed proteins and found that high-vigor seeds are enriched in proteins for sulfur amino acid metabolism, lipid and starch mobilization, protein synthesis (e.g., abundant translation initiation factors), and ABA signaling components [126]. These molecular functions were proposed as biomarker modules for seed vigor. Indeed, an earlier proteomic study in sugar beet identified specific proteins (e.g., late embryogenesis abundant proteins and detoxification enzymes) whose abundance predicted seed lot performance [126]. In *Arabidopsis*, proteomics of dry vs. primed seeds has similarly highlighted proteins involved in energy metabolism and desiccation tolerance that mark enhanced vigor [127]. Another notable study on alfalfa, comparative proteomics between primed and unprimed seeds, revealed upregulation of stress-protective proteins and metabolic enzymes in primed (high-vigor) seeds [128]. In maize, a 2D-DIGE proteomic analysis of five hybrids and their parents showed non-additive protein expression patterns underlying heterosis during germination [129]. Hundreds of proteins exhibited dominance or over-dominance expression in hybrids, particularly those related to ABA/GA hormone signaling, stress response, and protein folding, aligning with the enhanced germination vigor observed in hybrid seeds [129]. Such studies demonstrate that seed vigor is a polygenic trait manifested as broad shifts in the proteome, and they provide candidate protein markers (e.g., specific chaperones, antioxidant enzymes, and germination regulators) for breeding selection. Because seed vigor and deterioration are closely related to metabolic status, metabolite profiling has proven valuable for biomarker discovery. In hybrid rice, non-targeted metabolomics (GC–MS) on seeds from 16 different crosses (before and after storage) pinpointed several metabolites strongly associated with vigor loss [130]. Notably, galactose and gluconic acid levels rose significantly in aged seeds and showed a high negative correlation with germination percentages [130]. In other words, seeds accumulating more galactose (a product of reserve breakdown) tended to have poorer vigor, making these sugars robust metabolic biomarkers of aging [130]. Glycerol was another metabolite negatively correlated with germination in most hybrids [130]. Such markers enable rapid, quantitative vigor assessment: for instance, galactose and gluconic acid content can indicate seed lot quality and remaining shelf life. Consistent patterns have been observed in other species. An integrated metabolomic-transcriptomic study of wheat seeds (comparing an aging-tolerant vs. sensitive cultivar) found that the tolerant line suppressed galactose accumulation during storage, whereas the sensitive line showed hyperactivation of the galactose metabolism pathway under aging stress [131]. This suggests that excessive galactose (and related sugar flux) is causative in vigor reduction—a conclusion reinforced by the upregulation of galactose-pathway genes (galactokinases, galactinol synthases, etc.) specifically in low-vigor wheat seeds. Beyond sugars, other metabolite classes have been linked to early vigor: free amino acids and TCA-cycle intermediates (energy metabolites) tend to drop in low-vigor seeds [122], whereas antioxidant metabolites (e.g., flavonoids and tocopherols) often increase in seeds primed for stress tolerance [132]. For example, in the desert halophyte *Tamarix hispida*, a combined transcriptome–metabolome analysis revealed that flavonoid compounds (like quercetin derivatives) massively accumulate during germination and early seedling growth, presumably to enhance oxidative stress protection in extreme environments [132]. Thus, multi-omics studies across diverse species consistently highlight metabolic traits—efficient energy usage, controlled reserve mobilization, and robust antioxidant pools—as key biochemical signatures of seed vigor. Although less prominent than the other previously mentioned omics types, epigenomic analyses emerge in seed vigor research. DNA methylation and chromatin state differences between dormant and non-dormant seeds (e.g., at hormone biosynthesis genes) have been noted, and these may form heritable marks influencing germination speed [133,134]. Likewise, ionomic profiles (elemental composition) of seeds can reflect nutrient loading and stress history, potentially serving as additional indicators of seed quality [135]. As multi-omics datasets grow, researchers can cross-validate these diverse biomarkers and prioritize the most predictive ones for breeding and seed technology. Together, these multi-omics biomarkers provide a foundation for developing predictive seed-quality diagnostics and for integrating molecular vigor indices into breeding and seed technology pipelines.

## 5. Artificial Intelligence in Developmental Modeling

Advances in artificial intelligence (AI) are enabling precision modeling of early plant development, from automated image analysis of seedlings to virtual crop simulations. Modern AI approaches—including deep learning, reinforcement learning, and digital twin modeling—are being harnessed to extract hidden patterns, optimize growth protocols, and forecast genotype–environment (G × E) outcomes in ways previously unattainable. High-resolution imaging of seeds and seedlings, combined with deep learning (especially convolutional neural networks, CNNs), has revolutionized phenotyping at early growth stages [136]. Computer vision algorithms can now identify, segment, and quantify seedling traits with minimal human intervention, achieving both high throughput and accuracy [137]. For example, CNN-based tools like RootNav 2.0 have replaced tedious manual tracing of roots with fully automated analysis, extracting complex root system architectures (RSAs) from images despite challenges like overlapping roots and variable lighting [138]. In a wheat seedling assay, RootNav 2.0 achieved segmentation accuracy comparable to expert annotation while running 10× faster than previous semi-automatic methods [138]. Notably, the deep model generalized well—with minor retraining, it accurately traced roots in *Arabidopsis* seedlings on plates and Brassica in hydroponics, demonstrating the transferability of deep learning across species and imaging setups. Such flexibility is crucial for breeding programs that handle diverse germplasm [138]. Beyond roots, deep learning is accelerating the assessment of germination and early vigor. Modern CNNs can detect subtle visual cues of germination (radicle emergence, seed coat changes) from time-lapse images, enabling per-seed fate tracking at scale [139]. Genze et al. (2020) [139] developed a region-based CNN to monitor over 2400 seeds of maize, rye, and pearl millet throughout germination. Their model automatically identified each seed in Petri dish images and predicted whether it had germinated, with >94% mean average precision on hold-out tests. This level of accuracy surpassed manual counting and classical thresholding, allowing precise calculation of germination parameters like mean germination time and uniformity. Similarly, it was noted that deep CNNs handle variable lighting and occlusions far better than traditional vision, because the networks learn invariant feature representations. Indeed, CNN-based methods avoid the need for crop-specific tuning of image thresholds, which often plagued older systems (e.g., GERMINATOR and SVIS relied on fixed color/size filters) [139]. By automating germination scoring across different species and conditions, deep learning provides more objective and reproducible seed quality assessments. Deep learning also enables multi-dimensional seedling phenotyping. Recent studies combine color and depth (RGB-D) imaging with CNN models to capture 3D growth traits. Samiei et al. [140] first demonstrated that deep neural networks could quantify seedling emergence and growth in soil-less systems using simple RGB videos. To facilitate broader adoption, Couasnet et al. [141] released an open-source software package (GrowthData v1.0) for analyzing seedling growth from RGB-D sequences. Using these tools, researchers can now monitor seedling vigor kinetics (e.g., rate and uniformity of emergence) under realistic conditions (soil, variable moisture)—a breakthrough for seed lot evaluation and early vigor screening [141]. Indeed, uneven emergence is a major cause of yield loss, and these high-throughput imaging platforms allow breeders to quantify and select for more vigorous, uniform seedlings. Deep learning approaches are proving effective in the field as well. Lightweight CNN models have been tailored for aerial and proximal sensing of young crops. For instance, Zhang et al. [142] designed compact U-Net variants to segment rice seedlings in UAV multispectral images. Despite being much smaller networks, these models achieved high precision in differentiating rice seedlings from soil background, enabling accurate plant density and early growth assessments from drone data [142]. This is critical for precision agriculture—farmers can obtain emergence maps and detect gaps or stresses within days of sowing, allowing timely re-seeding or interventions. The adaptability of deep models to different data modalities (RGB, depth, multispectral) underscores their central role in next-generation phenotyping. A recent review by Weihs et al. [143] emphasizes that AI-driven image analysis has become indispensable for RSA and early development studies, noting its contributions and outlining future challenges. Taken together, deep learning is moving phenotyping from manual, low-throughput measurements to an era of automation and big data, where millions of datapoints on seedling growth can be collected and analyzed to inform breeding and management [144]. A wide range of real-world applications now demonstrate how different AI techniques—spanning convolutional neural networks (CNNs), recurrent architectures, and reinforcement learning—are being deployed across seed and seedling phenotyping pipelines. Table 3 summarizes key studies in this domain, highlighting the specific models, data modalities, and outcomes achieved.

While deep learning excels at perception (e.g., image-based trait extraction), reinforcement learning (RL) enables AI to make decisions in a trial-and-error manner to optimize a desired outcome [156]. In the context of early plant development, RL is emerging as a tool to discover optimal cultivation protocols and breeding strategies that would be difficult to find via intuition or one-factor-at-a-time experiments [157]. The power of RL lies in its ability to learn from interactions with an environment (real or simulated) by maximizing a reward function, which can represent outcomes like germination success, seedling biomass, or genetic gain [157]. One promising application is using RL to control and optimize growth conditions in real time. In greenhouse settings, managing climate (temperature, light, humidity, etc.) for seedlings is a complex control problem with dynamic plant responses [158]. Traditional climate controllers (e.g., PID or heuristic rules) are often suboptimal, especially under changing weather or for different crop stages [159]. RL agents, in contrast, can continuously adjust control actions (venting, heating, irrigation) based on feedback from the plants and sensors. Studies show that deep RL algorithms can outperform conventional model-based controllers by adapting to each developmental stage and even to different cultivars [160]. For instance, an RL agent trained in simulation was able to learn climate control policies that maintained crop growth while reducing energy use, something that would be hard to achieve with fixed rules [160]. Such agents essentially “learn” the optimal protocol (e.g., when to water or how to modulate light) through repeated experimentation in a safe virtual environment, and the learned policy can then be applied in the real greenhouse. Early results in lettuce and tomato greenhouses indicate RL can increase yield and resource-use efficiency simultaneously, adjusting to plant feedback in ways human operators might not anticipate [160]. As Internet of Things (IoT) sensor networks provide rich real-time data, the integration of RL in decision-support systems is poised to make greenhouse propagation more autonomous and climate-smart [161].

Reinforcement learning is also being applied at a higher decision level—to optimize breeding and management protocols. A notable example is the use of RL to design crop breeding programs. Younis et al. [162]. formulated the breeding process as a sequential decision-making problem (Markov Decision Process), where at each cycle the “agent” must choose which plants to select and cross [162]. By training an RL algorithm on simulated populations of maize (using real genomic data), they showed the AI breeder could achieve greater genetic gain over generations than standard genomic selection methods. In other words, the RL policy learned to balance short-term gains with maintaining genetic diversity for long-term improvement [162]. This approach is novel because it considers the breeding pipeline holistically, making coordinated decisions on crossing, selection intensity, and population size, rather than optimizing each step in isolation. The result was a more efficient accumulation of favorable alleles under resource and time constraints, illustrating how AI can help navigate the enormous search space of breeding strategies. While still in silico, these findings pave the way for AI-assisted breeding program design, where algorithms suggest crossing plans or selection schemes that human breeders might not intuitively devise. One emerging frontier is automating experimental protocols with reinforcement learning (RL). For example, one could envision an RL-driven robotic system that adjusts seed priming duration or temperature on the fly to maximize germination for specific seed lots—an approach supported by work on robotic RL in physical task automation [163,164]. Similarly, “active learning” algorithms can decide which phenotypic measurements to take next (and where/when) to most efficiently characterize seedling growth. The general trend is that reinforcement learning enables adaptive, closed-loop optimization, in contrast to static one-size-fits-all protocols. By continually learning from outcomes (e.g., seedling emergence, growth rates), RL-based systems promise to discover non-intuitive strategies for improving early development under variable conditions [165]. Integrating human knowledge (e.g., via human-in-the-loop RL or by shaping reward functions) can further ground these algorithms in practical reality. In summary, reinforcement learning brings a powerful paradigm for “self-optimizing” cultivation and breeding processes, pushing the boundaries of what can be achieved in early-stage plant development. Despite its potential, AI integration in seed and seedling phenotyping raises concerns related to interpretability, bias, accessibility, and ecological resilience [166,167]. One key concern is the transparency and interpretability of AI-driven decisions [168]. Deep learning models, especially those used for trait prediction or selection recommendations, often function as “black boxes”, making it difficult for breeders or agronomists to understand why a particular genotype or treatment was favored [169]. This lack of explainability can undermine trust and hinder informed decision-making in breeding programs. There is also a risk that algorithmic bias [170] and equity [171] in AI-driven applications could lead to suboptimal or unfair outcomes. For example, CNNs trained primarily on plate-based seedling images may perform poorly when applied to field-grown varieties, potentially disadvantaging more diverse or under-represented germplasm [172,173]. Moreover, the centralization of AI tools and data may exacerbate existing inequalities between large, tech-enabled institutions and smaller breeding programs or farmers in low-resource settings. The availability of high-quality imaging hardware, labeled training datasets, and computational infrastructure is often unevenly distributed. Ensuring equitable access to open-source tools, interoperable datasets, and capacity-building efforts is essential to avoid deepening technological divides. From a broader systems perspective, the automation of phenotyping and decision-making also raises questions about ecological resilience and over-optimization. If AI models consistently optimize short-term traits like rapid emergence or biomass under specific controlled conditions, they may inadvertently reduce genetic diversity or select against resilience traits that matter under real-world variability [174]. Embedding multi-objective optimization and human oversight into AI pipelines can help mitigate these risks. Finally, as reinforcement learning and digital twins become more embedded in real-time crop management, data privacy and cybersecurity must also be considered, particularly for IoT systems operating in commercial greenhouses or farms. Unauthorized access to breeding strategies, environmental sensor data, or control systems could pose both economic and biosecurity risks. In summary, responsible AI deployment in seed and seedling modeling requires not just technical performance but also transparency, fairness, inclusivity, and ecological foresight. Ethical guidelines, open governance frameworks, and interdisciplinary collaboration will be crucial to ensuring that the benefits of AI are widely and equitably shared across the agricultural innovation landscape.

## 6. Conclusions

Early plant development represents a highly sensitive and strategically important phase in which molecular, physiological, and environmental factors interact to determine later plant performance. This review demonstrates that recent advances in bioinformatics-driven omics analysis, chemometric modeling, and artificial intelligence have fundamentally expanded the capacity to analyze and manipulate seed germination, seedling establishment, and root system development in an integrated manner. By synthesizing evidence from CRISPR/Cas-mediated genome editing, microbiome-assisted seed and root engineering, and nanotechnology-enabled seed priming, this work shows that diverse intervention strategies consistently converge on a shared regulatory architecture linking hormonal balance, redox homeostasis, and root system plasticity. Recognizing this convergence provides a mechanistic basis for integrating otherwise disparate technologies into coherent early-stage improvement strategies. Importantly, this review positions early plant ontogeny as a tunable regulatory state governed by quantitative thresholds rather than as a fixed developmental sequence. In this context, artificial intelligence-driven phenotyping and modeling enable the prediction and optimization of early developmental trajectories across genotype-by-environment interactions, shifting early-stage selection from endpoint-based assessment toward trajectory-based control. Future progress will depend on the coordinated integration of molecular editing, biological priming, nanotechnology, and artificial intelligence within standardized and environmentally responsible frameworks. Establishing shared performance metrics, improving model interpretability, and ensuring accessibility across diverse agricultural systems will be essential for translating these advances from controlled environments to field-scale applications. Overall, the concepts synthesized here position early plant development as a central design target for next-generation crop improvement aimed at enhancing resilience, developmental uniformity, and yield stability under increasing environmental variability.

## Figures and Tables

**Table 1:** Regulatory Components Influencing Seed Germination and Stress Tolerance via Hormonal Pathways.

**Component**	**Role in Early Development**	**Stress Interaction**	**Reference**
DELLA proteins	Negative regulators of GA signaling; integrate stress signals	Accumulate under stress to suppress growth	[20]
DREB2B (*Arabidopsis*)	Negatively regulates seed vigor via ABA-responsive genes	Suppresses germination under drought stress	[21]
ABI5 phosphorylation	MAPK3-mediated loop enhances ABA response	Regulates ABI5 nuclear localization under stress	[22]
AtbZIP62 (*Arabidopsis*)	Enhances seed germination and cotyledon greening via ABA signaling	Promotes drought tolerance through positive regulation of ABA-responsive genes	[23]
GhWRKY6-like	Enhances germination rate and root length	Improves salt/drought tolerance via ABA & ROS regulation	[24]
GhWRKY27a	Represses germination and root growth	Enhances drought susceptibility via ABA and stomatal misregulation	[25]
GhWRKY46	Improves survival and biomass	Upregulates RD22, CBL10, and CPK3 for drought/salt tolerance	[26]
GhWRKY4	Enhances seedling stress resilience	Targets GhHDA8 to regulate drought/salt response genes	[27]
GhWRKY1-like	Promotes germination via ABA biosynthesis genes	Enhances drought tolerance by activating NCED gene family	[28]
GhWRKY17 (cotton)	Negatively regulates seed germination and ABA sensitivity	Reduces tolerance to drought and salt via ABA signaling and ROS modulation	[29]
GmWRKY16 (soybean)	Promotes seed germination and root growth under stress	Enhances drought and salt tolerance via ABA pathway activation	[30]
OsWRKY50 (rice)	Represses ABA-induced germination and seedling growth	Increases salt tolerance independently of ABA	[31]
TaWRKY24 (wheat)	Enhances germination and root elongation	Improves drought and salt tolerance by regulating proline, ROS, and ABA responses	[32]
GsWRKY20 (soybean)	Represses ABA sensitivity during germination	Enhances drought tolerance via ABA and epicuticular wax regulation	[33]
WRKY6 (*Arabidopsis*)	Positively regulates ABA signaling during germination	Enhances ABA response by repressing RAV1, modulating ABI genes	[34]
WRKY63 (ABO3)	Regulates seedling establishment and ABF2 expression	ABA hypersensitivity, reduced drought tolerance	[35]
WRKY57	Enhances ABA biosynthesis and sensitivity	Improves drought tolerance, upregulates RD29A, NCED3	[36]
TaWRKY71	Improves seed germination under ABA	Enhances salt and drought tolerance in *Arabidopsis*	[37]
CsWRKY46 (cucumber)	Enhances ABA sensitivity and germination rate	Improves salt and drought tolerance	[38]
MdWRKY30 (apple)	Enhances germination and cotyledon greening	Promotes tolerance to salt/osmotic stress in *Arabidopsis*	[39]
FvWRKY42 (strawberry)	Increases ABA sensitivity, improves germination under salt/drought	Enhances drought and salinity tolerance via ABA and antioxidant pathway regulation	[40]
GhPLATZ1 (cotton)	Accelerates germination under stress, reduces ABA content in dry seeds	Improves seedling establishment via modulation of ABA/GA/ethylene pathways	[41]
ClWRKY20 (watermelon)	Enhances ABA response during germination, improves stress gene expression	Enhances salt and chilling tolerance by coordinating ABA, ethylene, and jasmonate signals	[42]
VvWRKY13 (grapevine)	Activates ABA biosynthesis pathway genes	Delays germination and enhances drought-related ABA signaling	[43]
TaWRKY44 (wheat)	Improves germination under osmotic stress	Increases salt and drought tolerance via ROS scavenging and ABA/GA response	[44]
SmWRKY40 (Eggplant)	Promotes seed germination and root elongation	Enhances tolerance to salt via ABA signaling and antioxidant activation	[45]

**Table 2:** Overview of nanoparticle-based seed priming approaches in various crops, detailing priming objectives, doses, and effects on germination, seedling growth, physiological responses, and yield outcomes.

**Crop (NP Type)**	**Priming Objective**	**Dose**	**Germination Impact**	**Seedling Growth Impact**	**Biochemical/Physiological Mechanisms**	**Yield/Final** **Outcome**	**Reference**
Pea (Fe_3_O_4_ NPs)	Drought stress	75 ppm	↑ germination rate	↑ root length (+38%), ↑ leaf number (+24%)	↑ antioxidant status, ↑ Fe uptake	↑ yield under drought	[103]
Canola (CaO NPs)	Germination & vigor	75 ppm	↑ germination (+30%)	↑ seedling FW (+34%), ↑ leaf number (+16%), ↑ chlorophyll (+29%)	Improved emergence under PEG stress	↑ yield (+35%)	[103]
Tomato (Se NPs)	Biotic & abiotic stress	~75 ppm	↑ germination (+22%)	↑ biomass, ↑ survival under pathogen (+72.9%)	↑ resistance to *Phytophthora*, *Botrytis*	Enhanced field resistance	[98]
Pea (Ag NPs, bio)	Disease resistance	50–100 mg/L	No inhibition	Healthy seedlings	↓ fungal infection (93–95%), ↓ disease index (78–88%)	↓ damping-off	[99]
Rice (ZnO + Se)	Germination & yield	Not stated	↑ emergence, ↑ vigor	↑ leaf area, ↑ nutrient uptake	↑ chlorophyll, ↑ phenolics	↑ grain yield	[104]
Rice (ZnS NPs)	Vigor & antioxidant	50 µg/mL	↑ germination	↑ seedling length, ↑ dry weight	↑ SOD, APX, CAT; ↑ gene expression	↑ seedling vigor	[105]
Wheat (Fe_2_O_3_ NPs)	Iron biofortification	Not stated	↑ germination %, ↑ uniformity	↑ seedling vigor	↑ Fe accumulation in grain	↑ Fe content	[97]
Barley (Se NPs)	Growth stimulation	5–10 mg/L	↑ germinability, ↑ energy	↑ root/shoot length, ↑ thickness	Dose-dependent; >5 mg/L = oxidative stress	↑ root traits (5 mg/L)	[106]
Chickpea (Fe_3_O_4_ NPs)	Antioxidant priming	Not stated	↑ germination rate	↑ seedling development	↑ tocopherols, ↑ carotenoids	↑ oxidative stress tolerance	[97]
Alfalfa (Fe_3_O_4_ NPs, green)	Drought stress	20–60 mg/L	↑ germination (+22–25%)	↑ shoot elongation (+115%), ↑ root surface area (+20.5%)	↑ α-amylase, ↑ lateral roots	↑ drought resilience	[107]
Stevia (SiO_2_ NPs)	Vigor & enzyme	10 ppm	↑ germination (+106%)	↑ root DW (+283%), ↑ shoot DW (+169%)	↑ catalase, ↑ peroxidase	↑ early vigor	[108]
Maize (CuO NPs)	Drought tolerance	Not stated	↑ germination	↑ biomass, ↑ pigments	↑ RWC, ↑ grain number	↑ yield under drought	[97]
Sorghum (Ag NPs, green)	Disease & vigor	Not stated	↑ germination rate	↑ seedling survival	↓ damping-off	↑ emergence	[109]
Watermelon (Ag NPs, green)	Growth & yield	Not stated	↑ germination uniformity	↑ seedling vigor	Maintained fruit quality	↑ yield	[110]
Tomato (MWCNTs)	Nano-carbon priming	Not stated	↑ germination (~90%)	↑ early seedling vigor	↑ aquaporins, ↑ water uptake	↑ germination kinetics	[97]
Pearl Millet (Ag NPs)	Salinity stress	Not stated	↑ germination	↑ seedling vigor	↑ antioxidants, ↑ chlorophyll	↑ salinity tolerance	[111]
Moringa (ZnO NPs)	Vigor & phytochemicals	10 mg/L	↑ germination speed & uniformity	↑ root/shoot length	↑ antioxidants, ↑ phytochemicals	↑ bioactive profile	[112]

**Table 3:** Summary of AI applications in seed and seedling phenotyping across domains.

**Category**	**Focus**	**Model/Technique**	**Key Outcome**	**Reference**
Germination	Germination scoring in 5 crops	SeedGerm + ML	Matched expert scoring across multiple crops	[145]
Germination	Germination rate in wild rice	SGR-YOLO (CNN)	94–98% accuracy; <1% trait error	[146]
Germination	Tomato seed germination prediction	KNN	94% accuracy from image features	[147]
Germination	Germination in soil under stress	RT-DETR-SoilCuc (Transformer)	98.2% mAP; robust to real soil images	[148]
Germination	*Arabidopsis* germination with robotics	SPENCER + ML	8000 seeds phenotyped; high-throughput under stress	[149]
Seedling Growth	Growth stage detection (e.g., cotyledons)	CNN + LSTM	>90% stage classification accuracy	[140]
Seedling Growth	Elongation tracking with depth info	CNN + RGB-D	Depth improves trait detection at night	[150]
Seedling Growth	Seedling emergence/vigor under field-like conditions	GrowthData (RGB-D)	Tracks uniformity, rate under soil/moisture variability	[141]
Seedling Growth	Rice seedling density/gap mapping from UAV	Compact U-Net	Accurate early emergence detection via drone imaging	[142]
Seedling Growth	BBCH stage classification in rice	EfficientNetB4 CNN	99.4% stage classification accuracy	[151]
Seedling Growth	Grading cold-region maize seedlings	CNN	98.6% precision for seedling quality grading	[152]
Root Architecture	RSA extraction	RootNav 2.0 (CNN)	10× faster than manual; expert-level segmentation	[138]
Root Architecture	Hypocotyl trait segmentation	U-Net	Achieves human-level accuracy on RGB	[153]
Root Architecture	Cultivar classification via root traits	RF + SVM	86% accuracy in pea cultivar identification	[154]
Root Architecture	Root-based yield grade prediction (cucumber)	U-Net + ResNet50	F1 score = 0.86; yield grade predicted from root phenotypes	[155]

## Data Availability

The data that support the findings of this study are available from the corresponding author upon reasonable request. Schematic figures in this manuscript were created using BioRender and AI-based image generation tools (Nano Banana Pro). Nano Banana Pro was used to generate visual schematics from author-provided prompts reflecting predefined scientific concepts. All figure structures, terminology, mechanistic relationships, and final visual content were critically reviewed, edited, and approved by the authors to ensure scientific accuracy and compliance with journal standards.
